# Combined Transcranial Direct Current Stimulation and Thermotherapy for Managing Central Post-stroke Pain in a Patient With Severe Paretic Hemiplegia: A Case Report

**DOI:** 10.7759/cureus.78593

**Published:** 2025-02-05

**Authors:** Koichiro Hirayama, Takeshi Fuchigami, Shu Morioka

**Affiliations:** 1 Physical Medicine and Rehabilitation, Kishiwada Rehabilitation Hospital, Osaka, JPN; 2 Neurorehabilitation, Kio university, Nara, JPN; 3 Neurorehabilitation, Kio University, Nara, JPN

**Keywords:** central post-stroke pain, stroke, tdcs, thalamic pain, thermotherapy, upper extremity

## Abstract

Central post-stroke pain (CPSP) is one of the sequelae that significantly impacts the quality of life for stroke patients. This case report examines the effects of a combined intervention, which included thermotherapy using warm water and transcranial direct current stimulation (tDCS) in a woman in her 50s who experienced severe left hemiplegia and CPSP following stroke. After thermotherapy aimed at reducing pain, tDCS was applied to the primary motor cortex for two weeks. In addition, robotic therapy targeting shoulder and elbow joint movements was introduced as a form of self-training to complement the intervention. Post-intervention assessments revealed improvements in upper limb motor function and a reduction in pain intensity, as indicated by a decrease in the numerical rating scale scores from a pre-intervention range of 4 to 10 to a post-intervention range of 4.5 to 6. These results suggest that a combined approach centered on tDCS may promote pain management and functional recovery in patients with CPSP.

## Introduction

Central post-stroke pain (CPSP) is moderate to severe chronic pain, with a prevalence in stroke patients ranging from 1% to 12% [1]. It can significantly reduce the quality of life (QOL) of patients with stroke [2,3], making it a critical issue in clinical practice. It typically results from thalamic lesions following a stroke and is characterized by severe diffuse pain with low local specificity. Treatment options for CPSP are limited, and systematic reviews and meta-analyses of interventions for CPSP have shown that no treatments have demonstrated efficacy in large-scale studies [4]. The certainty of evidence supporting existing treatments for CPSP is very low. Therefore, an integrated approach combining multiple treatments is recommended to alleviate pain and improve the QOL in stroke patients with CPSP [5]. 

Transcranial direct current stimulation (tDCS) is a safe, noninvasive brain stimulation method that delivers a weak direct current through sponge electrodes placed on the scalp. This technique aims to regulate interhemispheric inhibition between the affected and unaffected hemispheres following stroke to promote functional recovery [6]. Although many previous studies on tDCS focused on modulating interhemispheric inhibition and cortical excitability, recent research has begun to explore the analgesic effects of CPSP [7]. Fregni et al. suggested that the less invasive the stimulation, the more effective it could be, and that increased local activation of the motor cortex is highly relevant to controlling central pain [8]. However, knowledge regarding the application of tDCS in patients with severe motor paralysis remains limited.

In patients with severe motor paralysis, sensory input and motor output are significantly restricted, which could exacerbate the impact of CPSP on their QOL. It is essential to accumulate clinical evidence on the therapeutic application of tDCS in such patients. Moreover, tDCS has the potential to contribute not only to pain relief but also to the recovery of motor function and sensory abilities, allowing for the evaluation of its combined effects. This single-case report details the application of tDCS in a stroke patient with CPSP and severe motor paralysis, examining its effectiveness in alleviating pain and improving motor function.

## Case presentation

Patient information

**Figure 1:**
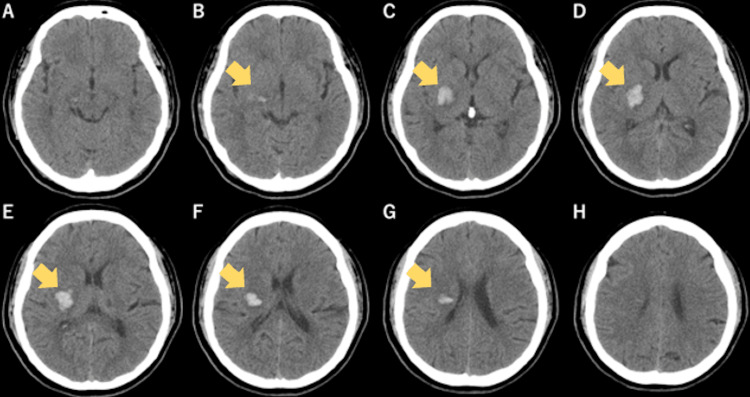
Initial CT imaging revealed a cerebral hemorrhage extending from the right putamen to the thalamus In the initial head CT scan immediately after onset, hemorrhage was observed in the right putamen, posterior limb of the internal capsule, and thalamus (B-G).

The patient was a right-handed woman in her 50s. She was transported to an acute care hospital after experiencing paresis in the left upper and lower limbs at work. Initial CT revealed a hemorrhage affecting the right putamen, posterior limb of the internal capsule, and thalamus (Figure 1). The day of onset was defined as X. Her medical history included only hypertension. During treatment at the acute care hospital, the hematoma did not increase in size; however, severe paralysis of the left upper and lower limbs persisted. On day X+17, she was transferred to our rehabilitation hospital during recovery. On admission, motor paralysis and sensory impairment persisted in the right upper and lower limbs

The Fugl-Meyer assessment for the upper extremity (FMA-UE) and lower (FMA-L) extremity both indicated severe impairment: the FMA-UE score was 4/66 points (shoulder, elbow, forearm = 4/wrist = 0/hand = 0/coordination and speed = 0), and the FMA-L score was 21/34 points. Muscle tone around the elbow joint was 1+ on the modified Ashworth scale, and both tactile and proprioceptive subtests of the stroke impairment assessment set (SIAS) scored 2 points. The motor activity log (MAL) reported 0 points for both amount of use (AOU) and quality of movement (QOM), indicating that the paretic hand was not used in daily activities. Cognitive function was intact, with a mini-mental state examination score of 30/30 indicating no significant cognitive impairment. The patient required assistance for all activities of daily living (motor functional independence measure (mFIM) = 42 points), and relied on a wheelchair for mobility. Based on these assessments, the rehabilitation plan for this patient included concurrent training in activities of daily living (ADL) and upper-limb function exercises aimed at achieving independent ADL.

On day X+31, the FMA-UE score improved to 13 points (shoulder, elbow, forearm = 12 / wrist = 0 / hand = 1 / coordination and speed = 0). The patient became independent in ADL using a wheelchair (mFIM = 71 points). The initial treatment goal prioritized independence in ADL, particularly focusing on walking and mobility, as this was considered essential for improving the patient’s QOL. Additionally, the burning sensations and other abnormal sensory symptoms reported by the patient shortly after onset were not severe enough to significantly disrupt daily activities. 

As a result of continued standard occupational therapy, the patient achieved independence in ADL with walking by day X+78 (mFIM = 81 points). However, the patient began reporting a burning sensation and pain radiating from the upper arm to the palm of the paretic limb. At this stage, the pain fluctuated significantly throughout the day, with a numerical rating scale (NRS) score ranging from 4 to 10, indicating very intense pain. The patient also experienced discomfort due to a mismatch between the perceived burning sensation and the cold skin temperature of the paretic limb.

To address these persistent and worsening sensory symptoms, thermal therapy aimed at alleviating upper limb pain was initiated on day X+87. This was followed by the stepwise application of tDCS for sensory impairment and upper limb dysfunction starting on day X+115. The timing of these interventions was carefully planned to address the sensory symptoms after the patient had achieved a certain level of functional independence in ADL.

From day X+78 to day X+87, the patient was sequentially prescribed carbamazepine, pregabalin, and duloxetine to manage the pain. However, the effects on pain relief were limited. After day X+87, duloxetine was continued as the primary medication.

Intervention protocol

The rehabilitation program during hospitalization included physical and occupational therapy, each conducted for one hour per day, seven days per week. The patient experienced pain in the paretic upper limb in response to various tactile stimuli, causing difficulty performing upper limb functional exercises requiring object manipulation or sensory input. Therefore, thermal therapy using warm water was initiated on day X+87 to alleviate pain in the paretic upper limb. 

**Figure 2:**
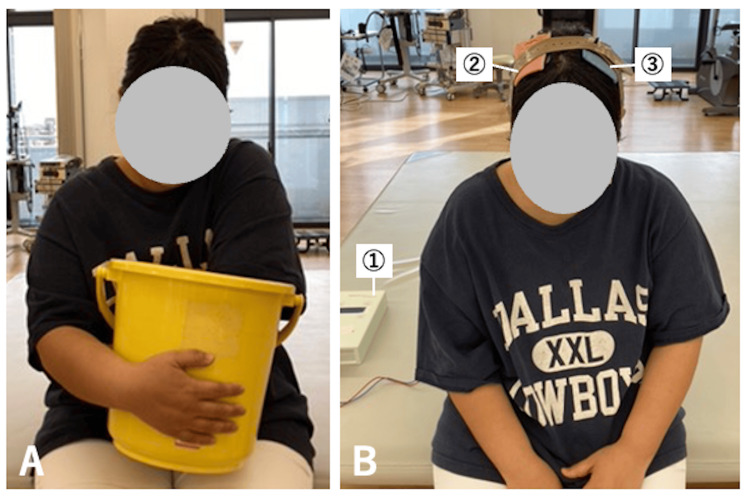
Thermal therapy using warm water and tDCS (A) Thermal therapy using warm water aimed to integrate the discrepancy between the burning sensation perceived in the paretic upper limb and the actual surface temperature of the paretic limb as perceived by the unaffected limb. The patient first perceived the temperature using the unaffected limb, then the paretic upper limb was immersed in a container filled with warm water at 40°C to 42°C for three minutes. (B) The tDCS setup included the following: ① The tDCS device that applies the stimulation; ② Anode electrode placed over the primary motor cortex of the affected hemisphere (C4); ③ Cathode electrode placed over the primary motor cortex of the unaffected hemisphere (C3). The electrodes were secured to the head using rubber bands and bandages. During the tDCS session, the patient remained seated at rest. Stimulation was applied for 20 minutes before the start of occupational therapy, after which the electrodes were removed, and the functional therapy session commenced. tDCS: Transcranial direct current stimulation

Thermal therapy involved immersing the paretic upper limb in a container (dimensions: width 34.3 cm × depth 31.5 cm × height 28.5 cm) filled with warm water at 40-42°C for three minutes (Figure 2). After the pain in the paretic upper limb had reduced sufficiently to allow for upper limb functional exercises (NRS: 5-7.25), combined therapy involving upper limb functional exercises and tDCS was initiated on day X+115. 

The tDCS was administered using a direct current stimulator (DC stimulator; NeuroConn, Munich, Germany). Stimulation was applied via surface sponge electrodes measuring 5 cm × 7 cm (35 cm²) (Figure 2). The electrodes were positioned with the anode placed over the primary motor cortex of the affected hemisphere (C4, according to the 10-20 system) and the cathode placed over the primary motor cortex of the unaffected hemisphere (C3). Each electrode was soaked in water and secured with a band. The intervention was performed for 20 minutes before each occupational therapy session, five times per week for two weeks. The stimulation intensity was set to 2.0 mA. Previous studies have shown these parameters to be safe [9,10].

Following tDCS, upper limb functional exercises consisted of task-oriented training that combined joint movements, such as shoulder flexion, elbow extension, and forearm supination, along with active sensory input for the fingers. Additionally, robotic therapy was continued as independent training, separate from occupational therapy interventions. In robotic therapy, the patient used the ReoGo-J upper limb rehabilitation device to perform self-training focused on flexion and extension movements of the shoulder and elbow joints for 30 minutes per day after physical and occupational therapy sessions. The ADL training exercises, such as dressing and bathing, were tailored to the patient’s level of independence.

Evaluation and analysis

Post-stroke upper limb paretic symptoms were evaluated using the FMA-UE [11]. The frequency of use of the paretic hand was assessed using the AOU and QOM scales of the MAL [12]. Both the FMA and the MAL have been translated into Japanese, and their reliability and validity have been verified. The pain was assessed using the subjective NRS. The patient recorded the NRS score to assess the overall pain in the paretic upper limb. To account for daily fluctuations in pain, NRS scores were recorded at four time points throughout the day (9:00, 12:00, 15:00, and 18:00), and the average score was calculated. Simple linear regression was employed to analyze fluctuations in the NRS over different periods, with time as the independent variable and the NRS score as the dependent variable, with regression lines calculated for each period (days 78 to 87, days 98 to 113, and days 115 to 129). Statistical analysis was performed using R Studio software ver. 2023.06.1+524 (Posit Software, Boston, MA, USA). Statistical significance was set at p < 0.05.

**Figure 3:**
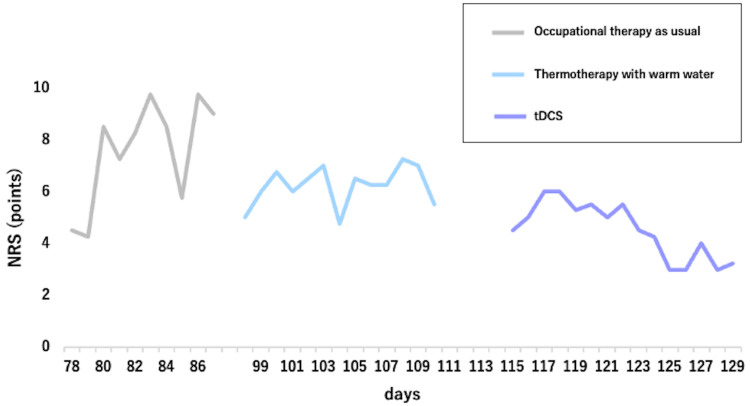
Fluctuations in NRS scores across three periods for the three different interventions The NRS scores during thermotherapy fluctuated more gradually than those during occupational therapy as usual, and decreased during tDCS. The periods of days 88 to 97 and 111 to 115 were excluded from the analysis as both periods were a transitional phase during which no specific interventions related to thermotherapy or tDCS were administered. The patient continued with standard occupational therapy, but no significant changes in their condition or treatment protocol were observed. NRS: Numerical rating scale, tDCS: Transcranial direct current stimulation

Comparisons of the pre- and post-intervention assessments are shown in Table 1 and Figure 3. The patient reported no discomfort or major adverse events associated with the tDCS sessions. The FMA-UE score improved from 13 points before the intervention to 21 points after the intervention. Following the intervention, the AOU score increased from 0.5 to 0.9, and the QOM score improved from 0.4 to 1.0. Comparing the NRS scores across different periods (Figure 3), the pre-intervention NRS scores ranged from 4 to 10 points, showing significant fluctuations, whereas the post-intervention scores improved to 4.5 to 6 points. Pain in the paretic upper limb gradually increased in NRS scores from 9:00 to 18:00 daily, reaching their peak at around 18:00 both before and after the intervention.

**Table 1:** Comparison pre- and post-intervention assessments FMA-UE: Fugl-Meyer assessment of the upper extremity; MAL: Motor activity log; SIAS: Stroke impairment assessment; FIM: Functional independence measure; QOM: Quality of movement; UE: Upper extremity, LE: Lower extremity

Parameters	Subscales	Pre X+87	X+115	Post X+130
FMA-UE (points)	Reflex activity	4/4	4/4	4/4
	Flexor synergy	8/12	9/12	9/12
	Extensor synergy	0/6	0/6	3/6
	Volitional movement mixing synergies	0/6	1/6	1/6
	Volitional movement with little or no synergy	0/6	0/6	0/6
	Normal reflex activity	0/2	0/2	0/2
	Wrist	0/10	0/10	0/10
	Hand	1/14	1/14	4/14
	Coordination/speed	0/6	0/6	0/6
MAL	Amount of use	0.5	0.5	0.9
	QOM	0.4	0.4	1.0
SIAS sensory function (points)	Light touch sensation in the UE	0/3	0/3	3/3
	Position sensation in the UE	3/3	3/3	3/3
	Light touch sensation in the LE	0/3	2/3	2/3
	Position sensation in the LE	2/3	2/3	2/3
FIM	Motor	81	84	86
	Cognitive	35	35	35

**Figure 4:**
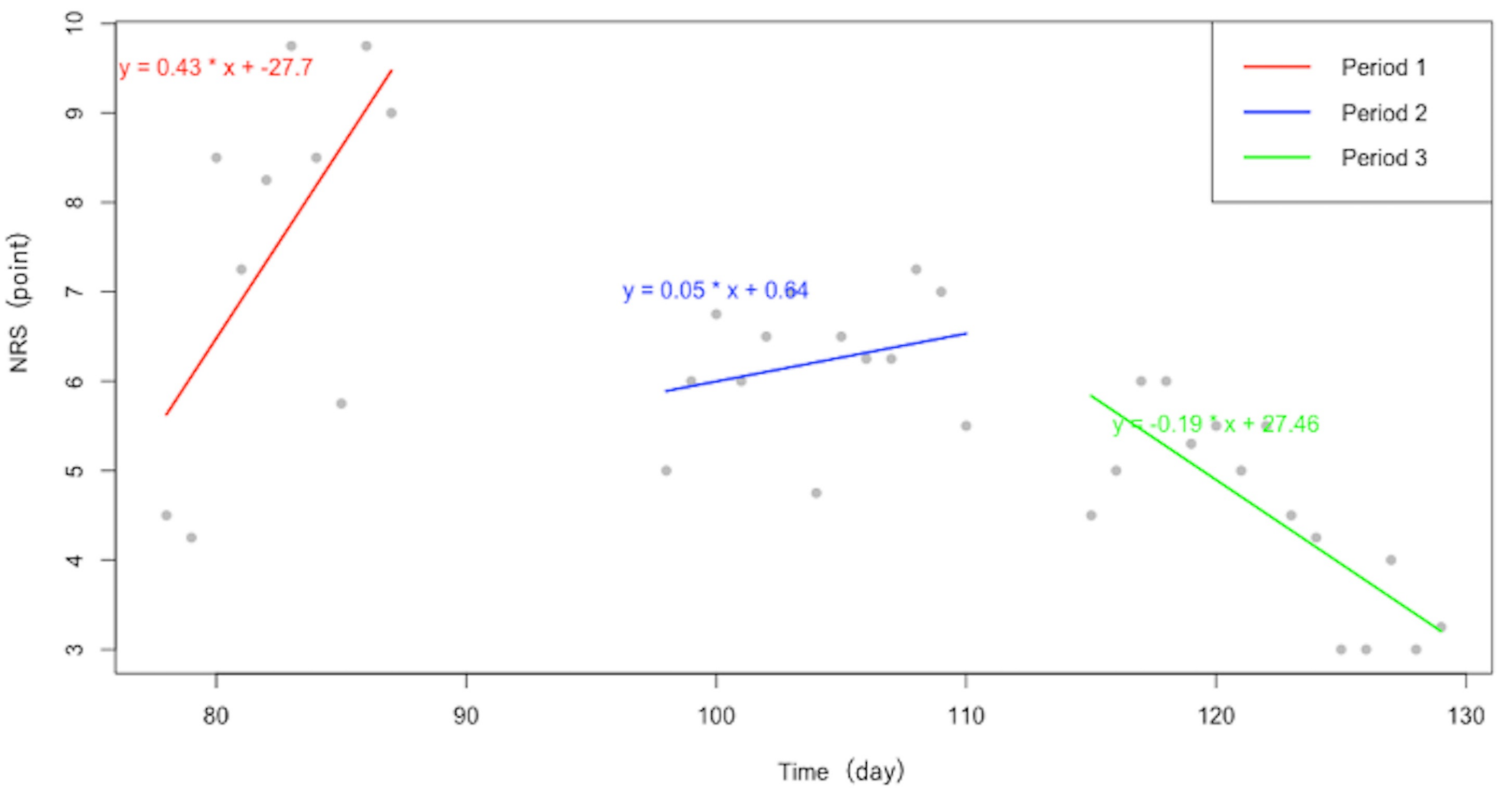
Regression lines and equations for each period Period 1: Occupational therapy as usual, Period 2: Thermotherapy with warm water, Period 3: tDCS NRS: Numerical rating scale, tDCS: Transcranial direct current stimulation

Figure 4 shows the regression lines and equations for each period. During the standard occupational therapy intervention (period 1), the regression equation was y = 0.43x - 27.7 (p = 0.05). For the period in which thermotherapy was applied (period 2), the equation was y = 0.05x + 0.64 (p = 0.37). During the period when tDCS was administered (period 3), the equation was y = -0.19x + 27.46 (p < 0.05). Based on the slopes of the regression lines, NRS scores tended to decrease during period 3, with a significant reduction in NRS scores only in period 3, when tDCS was applied.

## Discussion

The results of this single case suggest that tDCS may be effective in managing upper limb pain and promoting functional recovery in patients with stroke and CPSP. In this case, thermotherapy was initially applied for pain relief, and as pain in the paretic upper limb decreased, upper limb functional training in combination with tDCS became possible. Matsuda et al. reported that abnormal sensations caused by sensory mismatches can predict pain persistence [13]. Additionally, Lewis et al. indicated that the severity of pain is associated with alterations in body image, and greater pain is linked to reduced tactile sensation [14]. In this case, reduced tactile sensation and abnormal sensations might have contributed to the sensory mismatch and changes in body image, exacerbating the pain. Thermotherapy may have helped alleviate the pain by integrating the sensory mismatch between the perceived skin temperature of the paretic limb (felt by the intact limb) and the burning sensation caused by CPSP. 

Regarding the effects of tDCS on CPSP, a systematic review by Chen et al. demonstrated tDCS as an effective treatment modality [15]. Regarding the mechanisms of CPSP, Morishita et al. suggested that CPSP results from maladaptive network reorganization after a stroke, with the dysfunction of the primary motor cortex (M1) leading to an imbalance with the ventral posterolateral nucleus (VPL) of the thalamus [16]. This patient may have experienced pain due to an imbalance in interhemispheric inhibition and reorganization of networks involving the VPL caused by the right thalamic hemorrhage. The tDCS may have helped modulate the inhibitory relationship between the non-lesioned and lesioned hemispheres, contributing to the reduction of pain and improvement of upper limb function by adjusting the imbalance in the VPL.

However, this being a single case makes it difficult to generalize the results. Moreover, the combined use of thermotherapy and tDCS in this case prevents a complete separation of their individual effects, which should be acknowledged as a methodological limitation. Additionally, the possibility that the observed improvements were influenced by placebo effects or the natural course of recovery cannot be entirely excluded, and this should be considered a limitation of this case report. The current report did not include direct quantitative assessments of VPL activity or interhemispheric inhibition. As such, the proposed mechanism is based on prior research and remains a hypothesis. Nevertheless, the findings of this case suggest that a combined approach may be effective for treating CPSP. Further validation through large-scale studies is warranted.

## Conclusions

This case report suggests that tDCS can effectively reduce pain and improve upper limb function in patients with CPSP after stroke. Initial heat therapy allows for effective tDCS application, emphasizing the need for a combined therapeutic approach. Further large-scale studies are required to confirm these findings and establish tDCS as a viable treatment option for CPSP.
